# Supplementary material for: Thermodynamic Analysis of Chemical Hydrogen Storage: Energetics of Liquid Organic Hydrogen Carrier Systems Based on Methyl-Substituted Indoles
Source: Materials (Basel). 2023 Apr 6;16(7):2924. doi: 10.3390/ma16072924 (PMC10095710; doi:10.3390/ma16072924)
Supplement: Supplementary file 1 [file materials-16-02924-s001.zip › materials-2237644-supplementary.pdf]

materials MDPI

Supplementary Information

Table S1. Provenance, purity, and methods of purification of samples used in this work.

compound	CAS	suppliers	initial purity	final purity [a]
3-methyl-indole (cr)	83-34-1	Thermo Fisher Scientific	0.99	0.9998
1,2-dimethyl-indole (cr)	875-79-6	Sigma-Aldrich	0.99	0.9997

[a] Purification was performed by fractional sublimation. Mass fraction impurity determined by the gas chromatography.

Table S2. Reference values for $\Delta_f H^o_m$(298.15 K) used for thermochemical calculations at T = 298.15 K (p^o=0.1 MPa, in kJ·mol^{-1})

.structure	CAS	compound	Formula	$\Delta_f H^o_m$(298.15 K)	Ref
CH_3-CH_2-CH_3	74-98-6	propane	C_3H_6	-104.7 ± 0.5 (g)	[1]
CH_3-NH-CH_3	124-40-4	dimethylamine	C_2H_7N	-18.6 ± 0.8 (g)	[1]
CH_3-NH-CH_2-CH_3	624-78-2	N-methyl-ethanamine	C_3H_9N	-46.8 ± 2.0 (g)	[2]
$H_2C{=}CH_2$	74-85-1	ethene	C_2H_4	52.5 ± 0.4 (g)	[1]
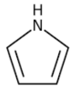	109-97-7	1-H-pyrole	C_4H_5N	108.3 ± 0.4 (g)	[1]
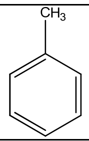	108-88-3	toluene	C_7H_8	50.4 ± 0.6 (g)	[1]
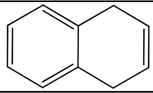	612-17-9	1,4-dihydro-naphtalene	$C_{10}H_{10}$	138.4 ± 1.6 (g)	[3]
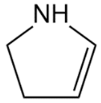	638-31-3	2-pyroline	C_4H_7	53.4±3.5 (liq)	Fig. S1
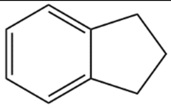	496-11-7	indane	C_9H_{10}	11.5±1.6 (liq)	[1]
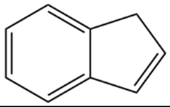	95-13-6	indene	C_9H_8	110.6±1.6 (liq)	[1]
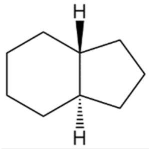	496-10-6	trans-hexa-hydro-indane	C_9H_{16}	-176.2±1.8 (liq)	[1]
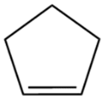	142-29-0	cyclopentene	C_5H_8	4.4±0.8 (liq)	[1]
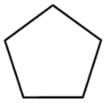	287-92-3	cyclopentane	C_5H_{10}	-105.1±0.8 (liq)	[1]
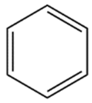	71-43-2	benzene	C_6H_6	49.0±0.6 (liq)	[1]

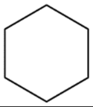	110-82-7	cyclohexane	C_6H_{12}	-156.4±0.8 (liq)	[1]
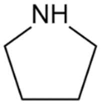	123-75-1	pyrrolidine	C_4H_9N	-41.0±0.8 (liq)	[1]

Table S3. The enthalpies of homodesmotic reactions (see Fig. 5) involving 3-methyl-indole $\Delta_r H^o_m$(g) at T = 298.15 K (p^o=0.1 MPa, in kJ·mol^{-1})

. $\Delta_r \boldsymbol{H}^o_m$**(g, 298.15 K)**	**R-A**	**R-B**	**R-C**
G4	92.0	-19.4	97.9
G3MP2	89.5	-21.0	95.2
CBS-APNO	90.8	-19.2	97.0

Table S4. Thermodynamics of phase transitions of indole derivatives (in kJ·mol^{-1})[a].

**Compounds**	$\Delta_{crII}^{crI} \boldsymbol{H}^o_m$ **at** T_{tr}	T_{fus}**/K**	$\Delta_{cr}^{l} \boldsymbol{H}^o_m$ **at** T_{fus}	$\Delta_{cr}^{l} \boldsymbol{H}^o_m$ [b]	$\Delta_{cr}^{g} \boldsymbol{H}^o_m$ [c]	$\Delta_{l}^{g} \boldsymbol{H}^o_m$ [d]
				**298.15 K**		
1	2	3	4	5	6	7
3-methylindole (phase II)	2.7±0.4 (316.8 K) [4]	368.0 [4]	11.6±0.5	11.4±1.2 [e]	81.4±0.6	70.0±1.3
3-methylindole (phase I)	2.7±0.4 (316.8 K)			1.9±0.4 [f]	81.2±0.7 [g]	69.8±0.6
2-methylindole	-	332.0 [S4]	14.8±0.1 [i] [S4]			
1,2-dimethylindole		330.7 [5]	14.9±1.0 [j]	13.3±1.1	83.2±0.7	69.9±1.3
2,3-dimethylindole		380.2 [6]	17.1±1.0 [j]	12.9±1.6	86.0±0.6	73.1±1.7 [k]

[a] Uncertainties in this table are expressed as expanded uncertainties at a level of confidence of 0.95, k=2).

[b] The experimental enthalpies of fusion $\Delta_{cr}^{l} H^o_m$ measured at T_{fus} were adjusted to 298.15 K with help of equation:

$\Delta_{cr}^{l} H^o_m$ (298.15 K)/(J·mol^{-1}) = $\Delta_{cr}^{l} H^o_m$ (T_{fus}/K) – ($\Delta_{cr}^{g} C^o_{p,m}$ - $\Delta_{l}^{g} C^o_{p,m}$)×[(T_{fus}/K) – 298.15 K] and heat capacity differences given in Table S3.

[c] From Table 4.

[d] Calculated as the difference of column 6 and 5 in this table.

[e] Sum of $\Delta_{crII}^{crI} H^o_m$ and $\Delta_{cr}^{l} H^o_m$ was adjusted from T_{fus} to T = 298.15 K using equation in footnote b.

[f] The phase transition enthalpy $\Delta_{crII}^{crI} H^o_m$ was adjusted from T_{tr} to T = 298.15 K using equation in footnote b.

[g] Calculated as follows: $\Delta_{cr}^{g} H^o_m$(crII) = (79.3±0.5) + (1.9±0.4) = (81.2±0.7) kJ·mol^{-1} with $\Delta_{cr}^{g} H^o_m$(crI) from Table 4.

[i] The Walden Constant [7] calculated for 2-methyl-indole according to equation: WC = $\Delta_{cr}^{l} H^o_m$ / T_{fus} = 14.8/332.0 = 0.045 J·mol^{-1}·K^{-1}.

[j] Calculate using the Walden Constant [7] calculated for 2-methyl-indole according to equation: $\Delta_{cr}^{l} H^o_m$ = WC × T_{fus} = 0.045×330.7 = 14.9±1.0 kJ·mol^{-1} for 1,2-dimethyl-indole and : $\Delta_{cr}^{l} H^o_m$ = WC × T_{fus} = 0.045×380.2 = 17.1±1.0 kJ·mol^{-1} for 2,3-dimethyl-indole.

[k] For comparison $\Delta_{l}^{g} H^o_m$ = (75.2±1.0) from Table 2.

In 1908 Paul Walden found that the ratio according to Eq. 8 can be considered as a constant (*W*alden´s Constant) [8]:

$$WC = \frac{\Delta_{cr}^{l}H_{m}^{o}}{T_{fus}} = \Delta_{cr}^{l}S_{m}^{o} = 56.5\ J{\cdot}K^{-1}{\cdot}mol^{-1} \quad (S1)$$

From a practical point of view and based on our experience, Eq. S1 can be easily adapted for calculations within a range of similarly shaped molecules. We have already observed similarity of *Walden´s Constants* for R-acetanilides with R = alkyl, F, Cl, Br, NO_2, NH_2, OH, OCH_3 [9] and for the for R-substituted benzamides [10]. We have found that for these series the *WC* for each series deviates from the "classic" value 56.5 $J{\cdot}K^{-1}{\cdot}mol^{-1}$ by about ± 10 $J{\cdot}K^{-1}{\cdot}mol^{-1}$. Such a "modified" *Walden's Constant* helps not only in evaluating the consistency of the experimental fusion data within a set of similarly structured compounds (see Table 12), but also the *Walden's rule* serves as a valuable tool for estimating missing fusion enthalpies of interesting organic compounds, provided that their fusion temperatures are available.

Table S5. Compilation of data on molar heat capacities $C_{p,m}^{o}$(cr or liq) and heat capacity differences $\Delta_{cr,l}^{g}C_{p,m}^{o}$ (in $J{\cdot}K^{-1}{\cdot}mol^{-1}$) at T = 298.15 K

Compounds	$C_{p,m}^{o}$(cr) [a]	$-\Delta_{cr}^{g}C_{p,m}^{o}$ [b]	$C_{p,m}^{o}$(liq) [a]	$-\Delta_{l}^{g}C_{p,m}^{o}$ [b]
3-methyl-indole	175.5	27.1	224.6	69.1
1,2-dimethyl-indole	202.2	31.1	231.6	70.8
1,3-dimethyl-indole	202.2	31.1	231.6	70.8
2,3-dimethyl-indole	160.2	24.8	282.7	84.1
3-methyl-indoline			247.4	74.8
1,2-dimethyl-indoline			258.5	77.8
1,3-dimethyl-indoline			258.5	77.8
2,3-dimethyl-indoline			281.9	83.9

[b] Calculated according to the procedure developed by Chickos et al. [11].

[b] Calculated according to the procedure developed by Acree and Chickos [12] by using experimental heat capacities $C_{p,m}^{o}$(cr or liq) from this table.

Transpiration method

Absolute vapour pressures were measured using the transpiration method [13,14]. The main idea of this method is to saturate the gas stream flowing over the sample and to determine the amount of compound transferred by the gas within a given time. Approximately 0.5 to 0.8 g of the sample is mixed with glass beads (diameter 1 mm) and placed in the thermostatted U-shaped saturator. The glass beads are needed to enlarge the contact area between gas and sample. A stream of nitrogen at a well-defined flow rate was passed through the saturator at constant temperature (± 0.1 K), and the transported material was collected in a cold trap. The amount of the condensed substance was determined by GC. The saturation vapour pressure p_i at each temperature T_i was calculated from the amount of condensate collected within a definite period of time:

$$p_i = m_i{\cdot}R{\cdot}T_a / V{\cdot}M_i\ ; \qquad V = (n_{N2} + n_i){\cdot}R{\cdot}T_a / P_a \quad (S2)$$

where V is the volume of the gas phase consisting of the n_{N2} moles of the carrier gas and n_i mole of gaseous compound under study (with the molar mass M_i) at the atmospheric pressure P_a and the ambient temperature T_a. The volume of the carrier gas V_{N2} was determined by the digital flow rate sensor from integration with a microcontroller. We used the Honeywell S&C - HAFBLF0200C2AX5 digital flow rate sensor with uncertainty at the level of 2.5 %. The flow rate of the nitrogen stream was also controlled by using a soap bubble flow meter (HP soap film flowmeter (model 0101-0113)) and optimized in order to reach the saturation equilibrium of the transporting gas at each temperature under study. The volume of the carrier gas V_{N2} was read from the digital flow sensor. The amount of the compound under investigation n_i in the carrier gas was estimated at each temperature using the ideal gas law.

Before starting the vapour pressure measurements, the sample was first pre-conditioned at 310-320 K (within about one hour) in order to remove possible traces of water. The saturator was then kept at 310-315 K (to remove possible traces of volatile compounds). To ensure that preconditioning was completed at the selected temperature, three samples were taken sequentially during sample rinsing and analyzed by GC. A constant vapour pressure at this temperature indicated that the transpiration experiments could begin. GC analysis of the transported material did not reveal any additional contamination. The absence of impurities and decomposition products was re-checked by GC analysis of the saturator content at the end of the entire series of experiments.

Thermodynamics of vaporisation/sublimation

Experimental vapour pressures measured in this work (as well as those from the literature) have been used to obtain the enthalpies of vaporisation using the following equation:

$$\Delta_{\mathrm{l}}^{\mathrm{g}} H_m^{\mathrm{o}}(T) = -b + \Delta_{\mathrm{l}}^{\mathrm{g}} C_{p,m}^{\mathrm{o}} \times T \quad \text{(S3)}$$

Experimental vapour pressures temperature dependences were also used to derive the vaporisation entropies at temperatures T by using the following equation:

$$\Delta_{\mathrm{l}}^{\mathrm{g}} S_m^{\mathrm{o}}(T) = \Delta_{\mathrm{l}}^{\mathrm{g}} H_m^{\mathrm{o}}/T + R \times \ln(p_{\mathrm{i}}/p^{\mathrm{o}}) \qquad \text{(S4)}$$

with p^{o} = 0.1 MPa. Coefficients a and b of Eq. (1), $\Delta_{\mathrm{l}}^{\mathrm{g}} H_m^{\mathrm{o}}(T)$ and $\Delta_{\mathrm{l}}^{\mathrm{g}} S_m^{\mathrm{o}}(T)$ values are collected in Table 1. The Eqs. S3-S4 are also valid for the treatment of vapour pressures measured over the solid sample, giving the standard molar enthalpy of sublimation $\Delta_{\mathrm{cr}}^{\mathrm{g}} H_{\mathrm{m}}^{\mathrm{o}}(T)$ and the standard molar sublimation entropy $\Delta_{\mathrm{cr}}^{\mathrm{g}} S_{\mathrm{m}}^{\mathrm{o}}(T)$. In this case in Eqs. S3-S4 we used the value $\Delta_{\mathrm{cr}}^{\mathrm{g}} C_{\mathrm{p,m}}^{\mathrm{o}}$ instead of the $\Delta_{\mathrm{l}}^{\mathrm{g}} C_{\mathrm{p,m}}^{\mathrm{o}}$-values (see Table S2). The combined uncertainties of the vaporisation enthalpies include uncertainties from the experimental conditions of transpiration. The uncertainties in vapour pressure and uncertainties due to the temperature adjustment to T = 298.15 K as described elsewhere [15,16]. The compilation of the standard molar enthalpies of vaporisation at the reference temperature T = 298.15 K, calculated according to Eq. (S3) is given in Table 1.

Temperature adjustments of sublimation/vaporisation enthalpies.

According to general practice, all thermochemical quantities must be represented at the reference temperature T = 298.15 K. Sublimation/vaporisation enthalpy derived from vapour pressure measurements are usually referenced to the average temperature, T_{av}, of the experimental range. In any case, they need to be adjusted to T = 298.15 K using a Kirchhoff´s Law. The isobaric heat capacity differences $\Delta_{\mathrm{cr,l}}^{\mathrm{g}} C_{\mathrm{p,m}}^{\mathrm{o}}$ are required for temperature adjustment of vaporisation/sublimation enthalpies according to Kirchhoff's rule. The simple and straightforward method was developed by Chickos and Acree [17]. They suggested an empirical way to assess $\Delta_{\mathrm{l}}^{\mathrm{g}} C_{\mathrm{p,m}}^{\mathrm{o}}$–values by equation:

$$\Delta_{\mathrm{l}}^{\mathrm{g}} C_{\mathrm{p,m}}^{\mathrm{o}}(298.15\ \mathrm{K}) = 0.26 \times C_{\mathrm{p,m}}^{\mathrm{o}}(\mathrm{liq},\ 298.15) + 10.58 \quad \text{(S4)}$$

where $C_{\mathrm{p,m}}^{\mathrm{o}}$(liq, 298.15 K)-data are of experimental origin or they can be also estimated according to the group-additivity procedure [11].

$$\Delta_{\mathrm{cr}}^{\mathrm{g}} C_{\mathrm{p,m}}^{\mathrm{o}}(298.15\ \mathrm{K}) = 0.15 \times C_{\mathrm{p,m}}^{\mathrm{o}}(\mathrm{cr},\ 298.15) + 0.72 \qquad \text{(S5)}$$

where $C_{\mathrm{p,m}}^{\mathrm{o}}$(cr, 298.15 K)-data are of experimental origin or they can be also estimated according to the group-additivity procedure [11].

Table S6. Vapor pressures, p_i, at different temperatures compiled from the literature [18], standard molar vaporisation enthalpies, $\Delta_l^g H_m^o$, and standard molar vaporisation entropies, $\Delta_l^g S_m^o$

T/ K[a]	p_i/ Pa[e]	$\Delta_l^g H_m^o(T)$/ [a] kJ·mol-1	$\Delta_l^g S_m^o(T)$/ J·K-1·mol-1
3-methylindoline. $\Delta_l^g H_m^o$(298.15 K) = (65.1±1.9) kJ·mol-1 $\ln(p/p_{ref}) = \frac{304.6}{R} - \frac{87406.2}{RT} - \frac{74.8}{R}\ln\frac{T}{298.15}$; p_{ref} = 1 Pa			
525	101325	48.1	91.7
329	47	62.8	127.0
330	47	62.7	126.2
2,3-dimethylindoline: $\Delta_l^g H_m^o$(298.15 K) = (67.1±3.9) kJ·mol-1 $\ln(p/p_{ref}) = \frac{313.3}{R} - \frac{90269.8}{RT} - \frac{77.8}{R}\ln\frac{T}{298.15}$; p_{ref} = 1 Pa			
523	101325	49.6	94.9
517	101325	50.0	96.9
508	101325	50.7	100.0
407	2666	58.6	113.8
398	2666	59.3	118.8
1,2-dimethyl-indoline $\Delta_l^g H_m^o$(298.15 K) = (60.8±2.3) kJ·mol-1 $\ln(p/p_{ref}) = \frac{306.0}{R} - \frac{84019.0}{RT} - \frac{77.8}{R}\ln\frac{T}{298.15}$; p_{ref} = 1 Pa			
501	101325	45.0	90.0
500	101325	45.1	90.3
378	3333	54.6	116.1
377	2400	54.7	114.0
376	2400	54.8	114.6
373	3333	55.0	119.1
368	1733	55.4	116.7
368	1733	55.4	116.7
367	1733	55.5	117.3
367	1733	55.5	117.3
315	47	59.5	125.0
314	47	59.6	125.9
1,3-dimethyl-indole, $\Delta_l^g H_m^o$(298.15 K) = (67.9 ± 1.2) kJ·mol-1 $\ln(p/p_{ref}) = \frac{304.0}{R} - \frac{89039.1}{RT} - \frac{70.8}{R}\ln\frac{T}{298.15}$; p_{ref} = 1 Pa			
533	101325	51.3	96.3
533	101325	51.3	96.3
532	101325	51.4	96.8
530	101325	51.5	97.3
529	101325	51.6	97.6
418	2400	59.4	111.1
413	2400	59.8	113.7
393	933	61.2	116.8
392	933	61.3	117.4
385	1067	61.8	122.6
353	80	64.0	122.0
336	47	65.2	130.3

335	47	65.3	131.1

1,3-dimethylindoline: $\Delta_l^g H_m^o$(298.15 K) = (60.0±2.3) kJ·mol^{-1}

$\ln(p/p_{ref}) = \frac{298.5}{R} - \frac{83158.9}{RT} - \frac{77.8}{R}\ln\frac{T}{298.15}$; p_{ref} = 1 Pa

523	101325	42.5	81.3
324	67	57.9	117.9
322	67	58.1	119.5

[a] Uncertainties of the vaporisation enthalpies are expressed as the expanded uncertainty (0.95 level of confidence, k = 2). They include uncertainties from the fitting equation, and uncertainties from temperature adjustment to T = 298.15 K. Uncertainties in the temperature adjustment of vaporisation enthalpies to the reference temperature T= 298.15 K are estimated to account with 20 % to the total adjustment.

Table S7. Correlation of vaporization enthalpies, $\Delta_l^g H_m^o$(298.15 K), of aromatic hydrocarbons with their Lee´s indices (J_{Lee})

.Compound	J_{Lee} [a]	$\Delta_l^g H_m^o$(298 K)$_{exp}$ kJ·mol^{-1}	$\Delta_l^g H_m^o$(298 K)$_{calc}$[b] kJ·mol^{-1}	Δ[c] kJ·mol^{-1}
tetraline	197.0	55.2	54.7	0.5
quinoline	210.3	59.3	58.3	1.0
iso-quinoline	214.1	59.5	59.4	0.1
2-methyl-quinoline	224.1	61.8	62.1	-0.3
3-methyl-quinoline	232.5	63.8	64.4	-0.6
4-methyl-quinoline	235.8	64.3	65.2	-0.9
6-methylquinoline	229.8	64.0	63.6	0.4
7-methylquinoline	231.4	63.7	64.1	-0.4
8-methyl-quinoline	225.2	62.0	62.4	-0.4
2-phenyl-quinoline	339.2	92.7	93.3	-0.6
2-phenyl-N-Me-indole	340.4	94.0	93.6	0.4
2-phenyl-N-Et-indole	356.0	98.3	97.8	0.5
1,2-dimethyl-indole	244.4		**67.6±1.0**	

[a] Kovats´s indices at 323 K, J_{Lee}, on the standard non-polar column SE-52 [19].

[b] Calculated using Eq. :

$\Delta_l^g H_m^o$(298.15 K) /(kJ·mol^{-1}) = 1.3 + 0.2712×J_{Lee} with (R^2 = 0.999)

with the assessed expanded uncertainty of ±1.0 kJ·mol^{-1} (0.95 level of confidence, k = 2)

[c] Difference between column 4 and 5 in this table.

Table S8. Difference in energetics between the *liquid-phase* reaction enthalpies, $\Delta_r H_m^o$(liq), and the *gas-phase* reaction enthalpies, $\Delta_r H_m^o$(g), of the hydrogenation of indole derivatives, at T = 298.15 K (p°=0.1 MPa, in kJ·mol^{-1})

Substituents	Reaction R-I	Reaction R-II	Reaction R-III	
	$\Delta(\Delta_r H_m^o)$	$\Delta(\Delta_r H_m^o)$	$\Delta(\Delta_r H_m^o)$	$\Delta(\Delta_r H_m^o)/H_2$ [a]
R = R_1 = R_2 = H	4.9	7.3	12.2	3.1
R = 2-CH_3; R_1 = R_2 = H	8.7	5.2	13.9	3.5
R = 3-CH_3; R_1 = R_2 = H	6.3	5.8	12.1	3.1
R = 2-CH_3; R_1 = H; R_2 = 3-CH_3	9.0	5.0	14.0	3.5
R = 2-CH_3; R_1 = CH_3; R_2 = H	6.9	8.4	15.3	3.8
R = H; R_1 = CH_3; R_2 = CH_3	6.9	8.3	15.2	3.9

[a] Difference in reaction enthalpy per mole H_2 calculated from data for Reaction R-III.

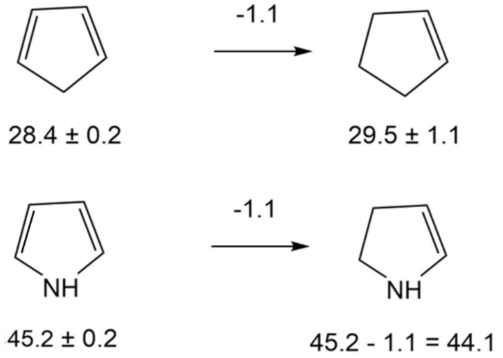

Figure S1. Calculation of vaporisation enthalpy of 2-pyroline:.

Line 1: difference between vaporisation enthalpies of 1,3-cyclopentadiene and cyclopentene;

Line 2: the difference from Line 1 was subtracted from vaporisation enthalpy of 1H-pyrrole.

Figures below molecules are $\Delta_l^g H_m^o$(298.15 K) taken from [1]. Figures above the arrows are differences in vaporisation enthalpies, $\Delta_l^g H_m^o$(298.15 K). All values are in kJ·mol^{-1}.

$\Delta_f H_m^o$(g, 298.15 K, 2-pyroline) = 97.5 kJ·mol^{-1} (calculated by G4(AT) in this work).

$\Delta_f H_m^o$(liq, 298.15 K, 2-pyroline) = 97.5- 44.1 = 53.4 kJ·mol^{-1} (see Table S2).

Table S9. Gas-phase standard molar thermodynamic properties of 2-methyl-indole and 2-methyl-H8-indole at T = 298.15 K.

**Compound**	$\Delta_f \boldsymbol{H}_m^o$	$\Delta_f \boldsymbol{S}_m^o$	$\Delta_f \boldsymbol{G}_m^o$	$\boldsymbol{S}_m^o$	$\boldsymbol{C}_{p,m}^o$
	kJ·mol^{-1}	J·K^{-1}·mol^{-1}	kJ·mol^{-1}	J·K^{-1}·mol^{-1}	J·K^{-1}·mol^{-1}
2-methyl-indole	121.6±1.5	-366.0	230.7	368.8	146.1
2-methyl-H8-indole	-99.3±2.2	-850.8	154.4	406.1	178.6

Computation of the thermodynamic properties for studied compounds in ideal gas state.

The conformer search was carried out by using Conformer Rotamer Ensemble Sampling Tool (CREST) by Grimmer utilizing metadynamic scan of the chemical compound, conformer and reaction space followed by optimization of the found conformers and rotamers at GFN2-xTB level of theory [20,21].

After the CREST stage, the geometries of all found conformers were further optimized and vibrational frequencies and methyl rotational potentials for molecules with CH_3- rotating top were computed using B3LYP hybrid density functional theory with the cc-pvtz(D3BJ) basis set [22,23] with the help of Gaussian 16 package [24]

Thus evaluated results correspond to approximation of «rigid rotor - harmonic oscillator». The most useful way to adjust the computed harmonic vibrational modes to the level of real molecules in gas phase is to scale them depending on the type of mode (stretching, deformational and so on). For complicated molecules distinguishing the type of vibrating mode is a challenging task and, in many cases, not possible. Therefore, we have divided the whole spectra into 4 regions: stretching N-H vibration (near 3500 cm^{-1}),

stretching C-H vibrations (2600 to 3100 cm^{-1}) and deformation vibrations in the intervals 1000 to 2000 cm^{-1} and below 1000 cm^{-1}. The values of scaling factors were evaluated by comparing the available in gas phase IR spectra [25] for indole, indoline and 2-methyl indoline with computational results. For 8H-indole only liquid phase IR spectrum is available for neat sample. No literature IR spectrum was found for 2-methyl-8H-indole. The results of comparison of scaled computed spectra with gas and liquid phase experimental spectrum is given in Figures S1-S4. In 2-methyl indoline and 2-methyl-8H-indole the methyl torsion was treated as a three-fold one-dimensional hindered rotor

.

Table S10. The gas-phase standard molar heat capacity, $C^{o}_{p,\mathrm{m}}$, of indole

T, K	$C^{o}_{p,m}$, [26] J K^{-1} mol^{-1}	$C^{o}_{p,m}$ (calc), J K^{-1} mol^{-1}	Δ [a]
298.15	120.9	120.9	0.0
300	121.7	121.9	0.2
320	130.4	132.9	2.5
340	139	143.1	4.1
360	147.3	152.8	5.5
380	155.4	161.9	6.5
400	163.2	170.6	7.4
420	170.8	178.8	8.0
440	178	186.7	8.7
460	184.9	194.2	9.3
480	191.5	201.4	9.9
500	197.8	208.3	10.5
520	203.8	214.9	11.1
540	209.5	221.3	11.8
560	215	227.4	12.4
580	220.2	233.4	13.2
600	225.2	239.1	13.9

[a] Difference between column 3 and 2 in this table.

Table S11. The gas-phase standard molar heat capacity, $C^{o}_{p,\mathrm{m}}$, of indoline

T, K	$C^{o}_{p,m}$, [26] J K^{-1} mol^{-1}	$C^{o}_{p,m}$ (calc), J K^{-1} mol^{-1}	Δ [a]
298.15	127.5	127.5	0.0
300	128.4	128.5	0.1
320	137.8	139.5	1.7
340	147.1	149.7	2.6
360	156.2	159.4	3.2
380	165.1	168.5	3.4
400	173.7	177.2	3.5
420	182.1	185.4	3.3
440	190.2	193.3	3.1
460	198.0	200.8	2.8
480	205.5	208.0	2.5
500	212.7	214.9	2.2
520	219.5	221.5	2.0
540	226.1	227.9	1.8
560	232.5	234.0	1.5
580	238.5	240.0	1.5
600	244.3	245.7	1.4

[a] Difference between column 3 and 2 in this table.

Table S12. The gas-phase standard molar heat capacity, $C^{o}_{p,\mathrm{m}}$, of 8H-indole

T, K	$C^{o}_{p,m}$, [26] J K^{-1} mol^{-1}	$C^{o}_{p,m}$ (calc), J K^{-1} mol^{-1}	Δ [a]
298.15	151.5	151.5	0.0
300	152.5	153.0	0.5
320	164.4	168.5	4.1
340	176.3	183.0	6.7
360	188.2	196.7	8.5
380	200.0	209.7	9.7
400	211.6	222.0	10.4
420	222.9	233.7	10.8
440	234.0	244.9	10.9
460	244.7	255.6	10.9
480	255.1	265.8	10.7
500	265.2	275.6	10.4
520	274.9	285.0	10.1
540	284.3	294.1	9.8
560	293.3	302.8	9.5
580	302.0	311.2	9.2
600	310.0	319.3	9.3

[a] Difference between column 3 and 2 in this table.

Table S13. The gas-phase standard molar heat capacity, $C^{o}_{p,\mathrm{m}}$, of 2Me-indole

T, K	$C^{o}_{p,m}$, [26] J K^{-1} mol^{-1}	$C^{o}_{p,m}$ (calc), J K^{-1} mol^{-1}	Δ [a]
298.15	146.1	146.1	0.0
300	147.0	147.1	0.1
320	156.5	158.1	1.6
340	165.9	168.3	2.4
360	175.1	178.0	2.9
380	184.1	187.1	3.0
400	192.8	195.8	3.0
420	201.3	204.0	2.7
440	209.4	211.9	2.5
460	217.2	219.4	2.2
480	224.7	226.6	1.9
500	231.9	233.5	1.6
520	238.8	240.1	1.3
540	245.4	246.5	1.1
560	251.8	252.6	0.8
580	257.8	258.6	0.8
600	263.7	264.3	0.6

[a] Difference between column 3 and 2 in this table.

Table S14. The gas-phase standard molar heat capacity, $C^{o}_{p,\mathrm{m}}$, of 2-Me-indoline

T, K	$C^{o}_{p,m}$, [26] J K^{-1} mol^{-1}	$C^{o}_{p,m}$ (calc), J K^{-1} mol^{-1}	Δ[a]
298.15	174	174.0	0.0
300	175	175.0	0.0
320	186.2	186.0	-0.2
340	197.1	196.2	-0.9
360	207.6	205.9	-1.7
380	217.8	215.0	-2.8
400	227.6	223.7	-3.9
420	236.9	231.9	-5.0
440	245.8	239.8	-6.0
460	254.3	247.3	-7.0
480	262.5	254.5	-8.0
500	270.2	261.4	-8.8
520	277.6	268.0	-9.6
540	284.6	274.4	-10.2
560	291.3	280.5	-10.8
580	297.8	286.5	-11.3
600	303.9	292.2	-11.7

[a] Difference between column 3 and 2 in this table.

Table S15. The gas-phase standard molar heat capacity, $C^{o}_{p,\mathrm{m}}$, of 2Me-8H-indole

T, K	$C^{o}_{p,m}$, [26] J K^{-1} mol^{-1}	$C^{o}_{p,m}$ (calc), J K^{-1} mol^{-1}	Δ[a]
298.15	178.6	178.6	0.0
300	179.8	180.1	0.3
320	193.0	195.6	2.6
340	206.1	210.1	4.0
360	219.2	223.8	4.6
380	232.1	236.8	4.7
400	244.8	249.1	4.3
420	257.3	260.8	3.5
440	269.3	272.0	2.7
460	281.0	282.7	1.7
480	292.4	292.9	0.5
500	303.3	302.7	-0.6
520	313.9	312.1	-1.8
540	324.1	321.2	-2.9
560	333.9	329.9	-4.0
580	343.3	338.3	-5.0
600	352.4	346.4	-6.0

[a] Difference between column 3 and 2 in this table.

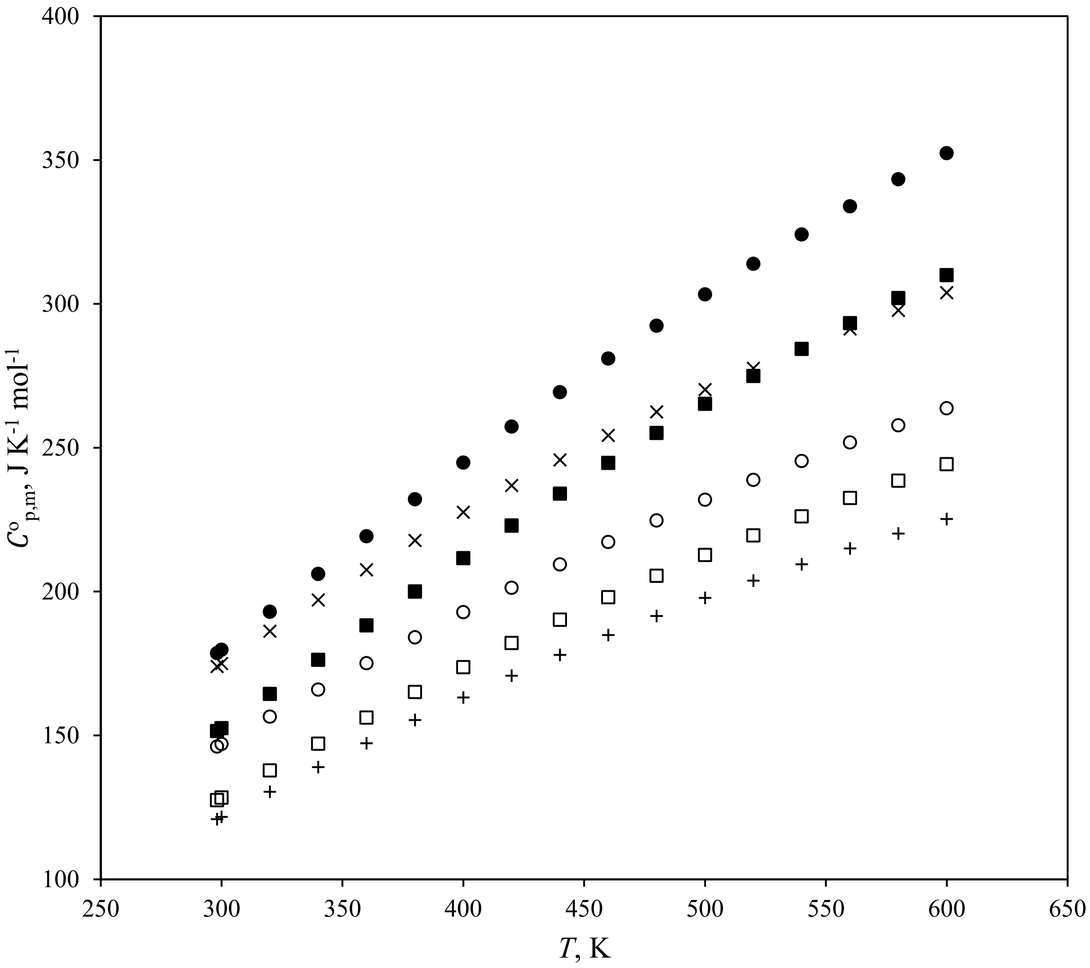

Figure S2. Temperature dependence of the gas-phase standard molar heat capacities:.

● - 2Me-8H-indole, ■ - 8H-indole, □ – Indoline, ○ - 2Me-indole, × - 2-Me-indoline, + indole.

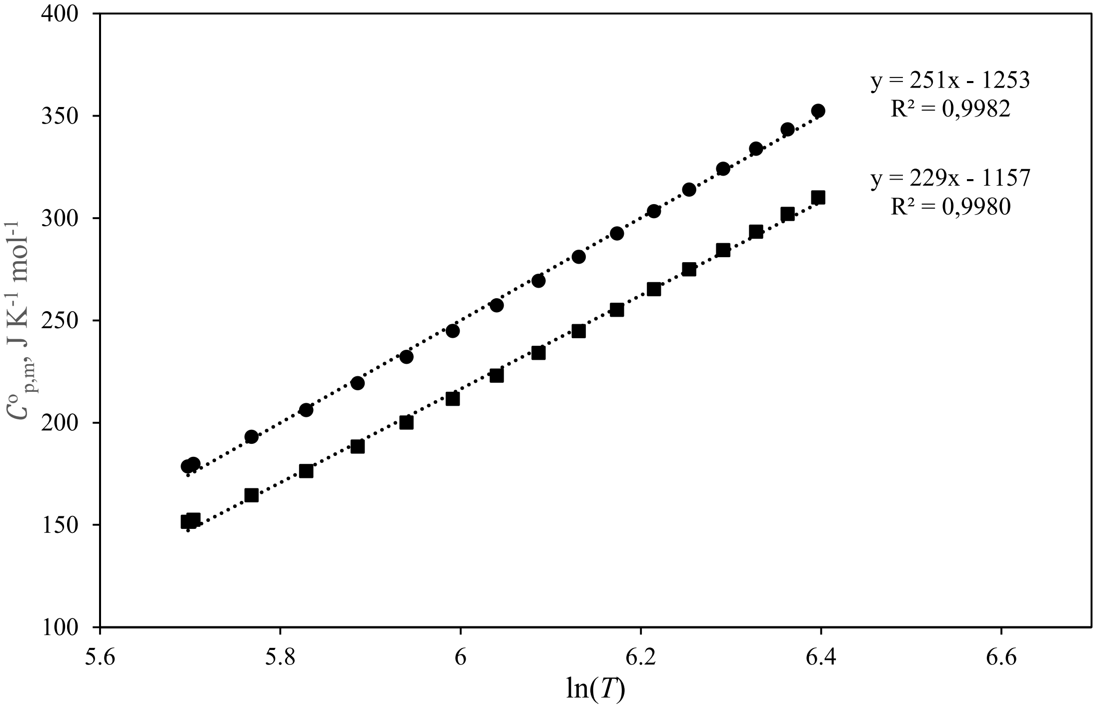

Figure S3. Dependence of the gas-phase standard molar heat capacity on $ln(T)$: ● - 2Me-8Hindole, ■ - 8H-indole

References

1. Pedley, J.B.; Naylor, R.D.; Kirby, S.P. *Thermochemical Data of Organic Compounds*; Chapman and Hall: New York, NY, USA, 1986; pp. 1–792.
2. Dorofeeva, O.V.; Filimonova, M.A.; Marochkin, I.I. Aliphatic Amines: A Critical Analysis of the Experimental Enthalpies of Formation by Comparison with Theoretical Calculations. *J. Chem. Eng. Data* **2019**, *64*, 5630–5647, doi:10.1021/acs.jced.9b00680.
3. Verevkin, S.P. Thermochemical Investigation on α-Methyl-Styrene and Parent Phenyl Substituted Alkenes. *Thermochim. Acta* **1999**, *326*, 17–25, doi:10.1016/S0040-6031(98)00585-1.
4. Safronov, S.P.; Vostrikov, S.V.; Samarov, A.A.; Wasserscheid, P.; Müller, K.; Verevkin, S.P. Comprehensive Thermodynamic Study of Substituted Indoles/Perhydro Indoles as Potential Liquid Organic Hydrogen Carrier System. *Fuel* **2023**, *331*, 125764, doi:10.1016/j.fuel.2022.125764.
5. Dean, B.D.; Truce, W.E. Oxidative Conversions of Sulfene-Cycloadducts from Azaheptafulvenes and from Tropone to 1,2-Disubstituted Indoles and 2-Aryl-Benzofurans, Respectively. *J. Org. Chem.* **1981**, *46*, 3575–3576, doi:10.1021/jo00330a050.
6. Sigma-Aldrich. Available Online: https://www.sigmaaldrich.com/ (accessed on 7 February 2023).
7. Abdelaziz, A.; Zaitsau, D.H.; Kuratieva, N.V.; Verevkin, S.P.; Schick, C. Melting of Nucleobases. Getting the Cutting Edge of "Walden's Rule". *Phys. Chem. Chem. Phys.* **2019**, *21*, 12787–12797, doi:10.1039/C9CP00716D.
8. Walden, P. Über Die Schmelzwärme, Spezifische Kohäsion und Molekulargrösse Bei Der Schmelztemperatur. *Z. Elektrotechnik Elektrochem.* **1908**, *14*, 713–724, doi:10.1002/bbpc.19080144302.
9. Nagrimanov, R.N.; Ziganshin, M.A.; Solomonov, B.N.; Verevkin, S.P. Thermochemistry of Drugs: Experimental and Theoretical Study of Analgesics. *Struct. Chem.* **2019**, *30*, 247–261, doi:10.1007/s11224-018-1188-z.
10. Verevkin, S.P.; Emel'yanenko, V.N.; Nagrimanov, R.N. Nearest-Neighbor and Non-Nearest-Neighbor Interactions between Substituents in the Benzene Ring. Experimental and Theoretical Study of Functionally Substituted Benzamides. *J. Phys. Chem. A* **2016**, *120*, 9867–9877, doi:10.1021/acs.jpca.6b10332.
11. Chickos, J.S.; Hosseini, S.; Hesse, D.G.; Liebman, J.F. Heat Capacity Corrections to a Standard State: A Comparison of New and Some Literature Methods for Organic Liquids and Solids. *Struct. Chem.* **1993**, *4*, 271–278, doi:10.1007/BF00673701.
12. Acree, W.; Chickos, J.S. Phase Transition Enthalpy Measurements of Organic and Organometallic Compounds and Ionic Liquids. Sublimation, Vaporization, and Fusion Enthalpies from 1880 to 2015. Part 2. C11-C192. *J. Phys. Chem. Ref. Data* **2017**, *46*, 013104, doi:10.1063/1.4970519.
13. Kulikov, D.; Verevkin, S.P.; Heintz, A. Determination of Vapor Pressures and Vaporization Enthalpies of the Aliphatic Branched C 5 and C 6 Alcohols. *J. Chem. Eng. Data* **2001**, *46*, 1593–1600, doi:10.1021/je010187p.

14. Verevkin, S.P.; Emel'yanenko, V.N. Transpiration Method: Vapor Pressures and Enthalpies of Vaporization of Some Low-Boiling Esters. *Fluid Phase Equilib.* **2008**, *266*, 64–75, doi:10.1016/j.fluid.2008.02.001.
15. Verevkin, S.P.; Sazonova, A.Y.; Emel'yanenko, V.N.; Zaitsau, D.H.; Varfolomeev, M.A.; Solomonov, B.N.; Zherikova, K. V. Thermochemistry of Halogen-Substituted Methylbenzenes. *J. Chem. Eng. Data* **2015**, *60*, 89–103, doi:10.1021/je500784s.
16. Emel'yanenko, V.N.; Verevkin, S.P. Benchmark Thermodynamic Properties of 1,3-Propanediol: Comprehensive Experimental and Theoretical Study. *J. Chem. Thermodyn.* **2015**, *85*, 111–119, doi:10.1016/j.jct.2015.01.014.
17. Chickos, J.S.; Acree, W.E. Enthalpies of Vaporization of Organic and Organometallic Compounds, 1880–2002. *J. Phys. Chem. Ref. Data* **2003**, *32*, 519–878, doi:10.1063/1.1529214.
18. SciFinder—Chemical Abstracts Service. Available Online: http://scifinder.cas.org/ (accessed on 7 February 2023).
19. Vassilaros, D.L.; Kong, R.C.; Later, D.W.; Lee, M.L. Linear Retention Index System for Polycyclic Aromatic Compounds. *J. Chromatogr. A* **1982**, *252*, 1–20, doi:10.1016/S0021-9673(01)88394-1.
20. Pracht, P.; Bohle, F.; Grimme, S. Automated Exploration of the Low-Energy Chemical Space with Fast Quantum Chemical Methods. *Phys. Chem. Chem. Phys.* **2020**, *22*, 7169–7192, doi:10.1039/C9CP06869D.
21. Grimme, S. Exploration of Chemical Compound, Conformer, and Reaction Space with Meta-Dynamics Simulations Based on Tight-Binding Quantum Chemical Calculations. *J. Chem. Theory Comput.* **2019**, *15*, 2847–2862, doi:10.1021/acs.jctc.9b00143.
22. Rappoport, D.; Furche, F. Property-Optimized Gaussian Basis Sets for Molecular Response Calculations. *J. Chem. Phys.* **2010**, *133*, 134105, doi:10.1063/1.3484283.
23. Grimme, S.; Ehrlich, S.; Goerigk, L. Effect of the Damping Function in Dispersion Corrected Density Functional Theory. *J. Comput. Chem.* **2011**, *32*, 1456–1465, doi:10.1002/jcc.21759.
24. Frisch, M.J.; Trucks, G.W.; Schlegel, H.B.; Scuseria, G.E.; Robb, M.A.; Cheeseman, J.R.; Scalmani, G.; Barone, V.; Petersson, G.A.; Nakatsuji, H.; et al. Gaussian 16, Revision C.01 Gaussian, Inc., Wallingford CT. 2016.
25. NIST Chemistry WebBook. Available Online: https://webbook.nist.gov/chemistry/ (accessed on 7 February 2023).
26. Konnova, M.E.; Li, S.; Bösmann, A.; Müller, K.; Wasserscheid, P.; Andreeva, I. V.; Turovtzev, V. V.; Zaitsau, D.H.; Pimerzin, A.A.; Verevkin, S.P. Thermochemical Properties and Dehydrogenation Thermodynamics of Indole Derivates. *Ind. Eng. Chem. Res.* **2020**, *59*, 20539–20550, doi:10.1021/acs.iecr.0c04069.
